# Bound Water as a Reinforcing Element for Ultra‐Strong Polyacrylamide

**DOI:** 10.1002/advs.76537

**Published:** 2026-07-13

**Authors:** Sirawit Pruksawan, Nannan Li, Zehuang Lin, Jianwei Zheng, Terence Jun En Loh, Yi Ting Chong, Junhua Kong, Xunan Hou, Xian Jun Loh, Chaobin He, FuKe Wang

**Affiliations:** ^1^ Agency for Science, Technology, and Research (A*STAR) Institute of Materials Research and Engineering (IMRE) Singapore Republic of Singapore; ^2^ Agency for Science, Technology, and Research (A*STAR) Institute of High Performance Computing (IHPC) Singapore Republic of Singapore; ^3^ Department of Materials Science and Engineering National University of Singapore (NUS) Singapore Republic of Singapore; ^4^ Nanyang Polytechnic (NYP) Singapore Republic of Singapore

**Keywords:** bound water, flame retardant, linear water cluster, polyacrylamide, reinforcing element, self‐extinguishing polymer, super strong polymer

## Abstract

Water is generally considered a plasticizer that weakens polymer mechanical properties. Here, we show that in polyacrylamide (PAM), water plays distinct roles depending on its state in the polymer matrix: free water plasticizes PAM into a soft gel, whereas bound water forms strong PAM–water–PAM hydrogen‐bond networks that surpass direct PAM–PAM interactions, enabling ultra‐strong PAM. Confined bound water reinforces the polymer matrix, increasing the tensile modulus from ∼0.6 to 9.2 GPa and the tensile strength from 2.7 to 140 MPa, while also yielding record‐high flexural (197 MPa) and compressive (178 MPa) strengths. Molecular dynamics simulations indicate that bound water organizes into linear nanoclusters that dynamically couple polymer chains, maximizing interchain cohesion and enabling efficient energy dissipation under stress. The resulting material surpasses many high‐performance polymers, including polyamide‐imide (PAI), polyether ether ketone (PEEK), and polyimide (PI), and exhibits exceptional flame resistance (limiting oxygen index ∼46). These findings establish bound water as an intrinsic structural enhancer, redefining its role in polymer reinforcement and providing a new design strategy for high‐performance synthetic polymers.

## Graphical abstract

Rather than acting as a conventional plasticizer, bound water serves as a reinforcing element in polyacrylamide by constructing strong PAM–water–PAM hydrogen‐bond networks. This water‐mediated architecture delivers ultra‐high mechanical strength and exceptional flame resistance, redefining the role of water in polymer reinforcement.

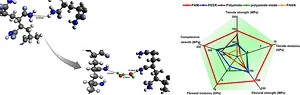

## 1 Introduction

Water is a critical structural element in many biological load‐bearing materials such as bone, tendon, cartilage, and nacre [1, 2, 3, 4, 5]. In these systems, water molecules form extensive hydrogen‐bond (H‐bond) and electrostatic interaction networks between proteins, polysaccharides, and mineral phases. These dynamic water‐mediated bridges distribute mechanical loads, dissipate energy through reversible bond breakage, and enable rapid structural recovery [6, 7, 8]. These synergies help biological structures achieve exceptional combinations of strength, stiffness, and toughness that remain unmatched by most synthetic materials [9, 10].

On the contrary, water is widely considered detrimental to the mechanical performance of synthetic polymers like nylon, epoxy, polyimide (PI), and so on [11]. Moisture uptake in these hydrophilic or partially hydrophilic polymers typically disrupts cohesive secondary interactions, lowers the glass transition temperature via plasticization, and thus reduces stiffness and strength [12, 13, 14]. However, a recent growing research is challenging this conventional view [15, 16, 17, 18]. These studies suggest that water can actively govern polymer structure and mediate polymer–polymer interactions, rather than acting merely as a passive plasticizer. This new understanding of water–polymer interactions is emerging as an increasingly important area of research for the development of advanced soft materials, hydrogels, and polymers. In light of this, a fundamental question arises: can water be harnessed as an enhancing structural element in synthetic polymers, as observed in natural systems?

Inspired by the constructive role of water in biological systems, we hypothesize that polyacrylamide (PAM), a synthetic polymer rich in protein‐like amide groups, can interact with water in a fundamentally different manner from conventional polymers. Our molecular dynamics (MD) simulations reveal that the role of water in PAM strongly depends on its state within the polymer matrix. In PAM containing large amounts of free water, water exhibits the conventional plasticizing effect, resulting in a soft gel material (Figure 1a). In contrast, removal of free water while retaining bound water, water functions as a reinforcing molecular bridge that forms strong PAM–water–PAM hydrogen‐bond networks surpassing the strength of direct PAM–PAM interactions in anhydrous PAM. This bound‐water‐mediated interaction enables the formation of ultra‐strong PAM with exceptional mechanical performance (Figure 1b). Upon complete removal of water, the PAM network becomes brittle due to the loss of these dynamic hydrogen‐bond bridges (Figure 1c).

**FIGURE 1:**
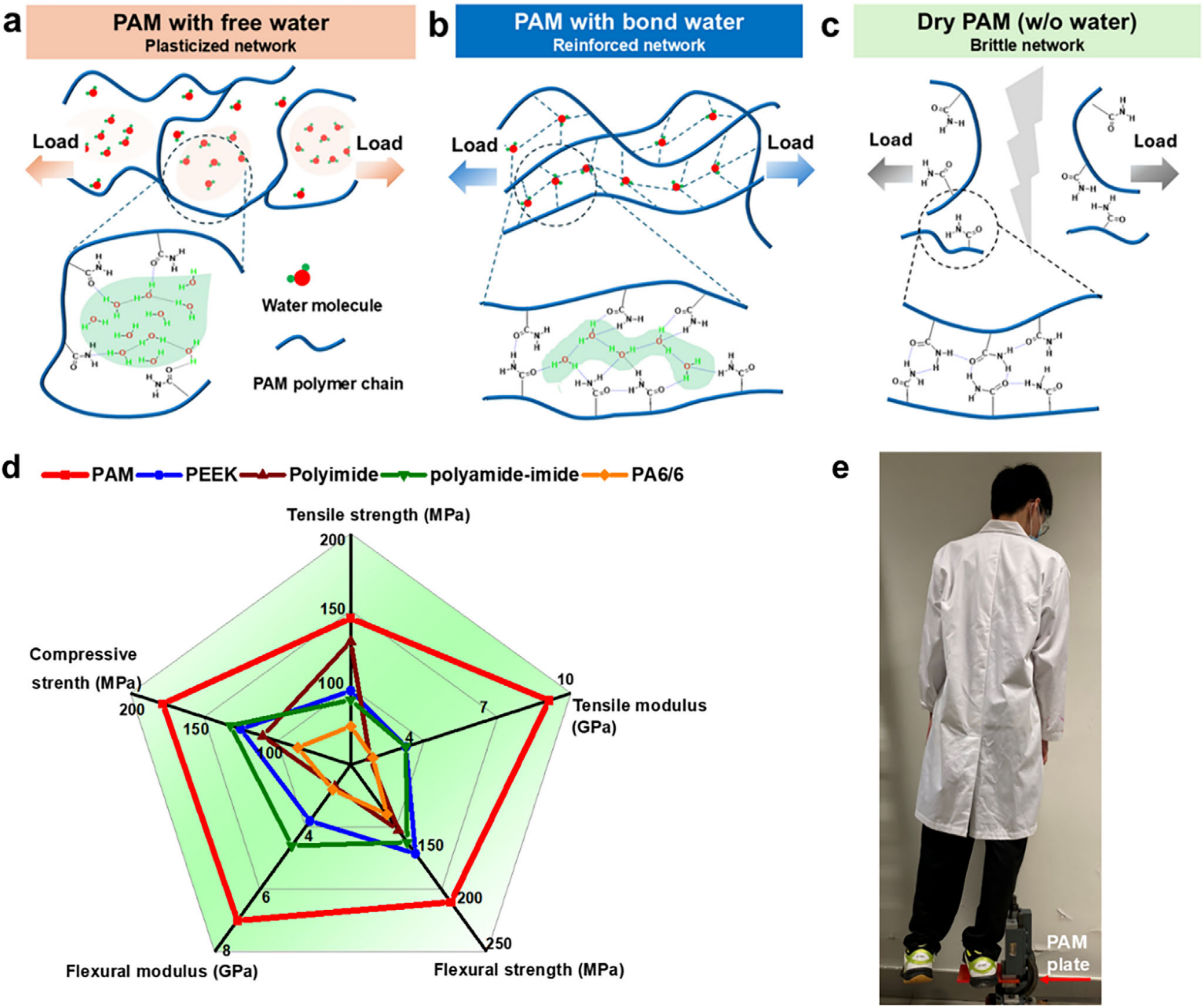
Conceptual schematic illustrating the roles of water in PAM networks: (a) PAM containing excess free water, where water acts as a plasticizer, (b) PAM containing bound water, where water functions as a reinforcing molecular bridge, and (c) anhydrous PAM without water‐mediated hydrogen‐bond bridging gives a brittle structure. (d) Mechanical performance of PAM with bound water compared to other available high‐performance polymers, including PEEK, polyimide, polyamide‐imide, and polyamide 6/6 (PA 6/6). (e) A 5‐mm‐thick PAM plate supports the weight of an adult man without failure or bending, demonstrating its exceptional mechanical strength.

Experimental results validate the simulation results and reveal a significant mechanical enhancement of PAM arising from bound water. PAM containing bound water achieves one of the highest combinations of stiffness and strength reported for polymer systems, including a tensile modulus exceeding 9 GPa, a tensile strength above 140 MPa, a flexural strength of 197 MPa, a flexural modulus of 7.0 GPa, and a compressive strength of 178 MPa (Figure 1d)—far exceeding typical high‐performance polymers like polyamide‐imide (PAI), polyether ether ketone (PEEK), and PI (Figure S1). Beyond mechanical reinforcement, the bound water also enhances the polymer's self‐extinguishing capability. Upon exposure to flame, the confined water rapidly vaporizes, absorbing heat and generating an internal porous structure that acts as a thermal and oxygen barrier, enhancing the self‐extinguishing capability of PAM. These findings redefine the role of water in high‐performance polymers and provide a new design strategy in which water is harnessed as a functional structural element rather than a mechanically weakening factor.

## 2 Results and Discussion

### 2.1 Dynamic Hydrogen Bond Network Leading to Exceptional Mechanical Reinforcement

Water is conventionally regarded as a plasticizer that weakens polymers, yet in polyacrylamide (PAM), it can be harnessed as a functional structural element. To investigate this, PAM samples with controlled water states were prepared via DLP 3D printing and stepwise dehydration. The printed PAM samples were dehydrated under a controlled thermal ramp to 85°C, followed by vacuum drying, removing free water while preserving strongly hydrogen‐bonded water. This dehydration process was maintained until the specimens reached a constant mass plateau representing the complete removal of free water and defining the equilibrium bound‐water fraction of the network. Long‐term mass stability confirmed the complete elimination of free water, consistently yielding PAM with ∼11 wt.% water, which corresponds to the non‐evaporable bound water.

The impact of bound water on mechanical performance is striking. Fully dried PAM exhibited a tensile strength of 2.73 MPa and modulus of 0.63 GPa (Figure S2), whereas PAM with bound water only achieved a tensile modulus of 9.2 GPa, tensile strength of 140 MPa, flexural strength of 197 MPa, flexural modulus of 7.0 GPa, and compressive strength of 178 MPa (Figure 1d; Tables S1–S3)—values surpassing most high‐performance polymers such as PAI, PEEK, and PI [19, 20, 21, 22], and approaching structural composites and metals (Figure S1). The material also sustained extreme loading: as shown in Figure 1e, a 5 mm‐thick PAM plate supported a 60 kg load without breaking. Furthermore, a silicone‐coated PAM tripod (0.5 mm‐thick top and 2 mm‐thick legs) held a 6 kg steel block while submerged in a water bath for five days, showing no significant structural deformation and a minimal weight gain of 0.84% (Figure S3). This result indicates that the challenges associated with the hydrophilic nature of PAM can be successfully addressed through surface coatings, enabling practical applications in moist environments. These findings suggest that bound water in PAM renders the polymer‐polymer interactions into a robust polymer‐water‐polymer bridging architecture, enabling mechanical performance that rivals conventional structural materials and highlighting a new paradigm where water acts as a reinforcing rather than a weakening element.

### 2.2 Acrylamide Concentration Impact and Unique Advantage of 3D Printing in Fabrication of High‐Performance PAM

PAM is conventionally synthesized by solution radical polymerization of acrylamide (AM) at concentrations typically ≤ 20 wt.%, because the formation of high‐molecular‐weight PAM during polymerization rapidly increases the viscosity of the reaction medium and, together with the exothermic nature of the reaction, complicates mixing, heat dissipation, and subsequent purification [23, 24, 25]. To probe these effects, we performed photo‐differential scanning calorimetry (Photo‐DSC) with lithium phenyl‐2,4,6‐trimethylbenzoylphosphinate (LAP) as photoinitiator (Figure 2a) across AM concentrations from 30 to 65 wt.%. Elevated AM concentrations sharply accelerated photopolymerization, with reaction enthalpies increasing from 211 to 528 J g^−1^ (Figure S4). This pronounced exothermicity heightens the risk of auto‐acceleration [26] and complicates polymerization control, yielding non‐uniform porous networks under standard photopolymerization conditions as illustrated in Figure 2b and ultimately poor mechanical performance (Figure 2c).

**FIGURE 2:**
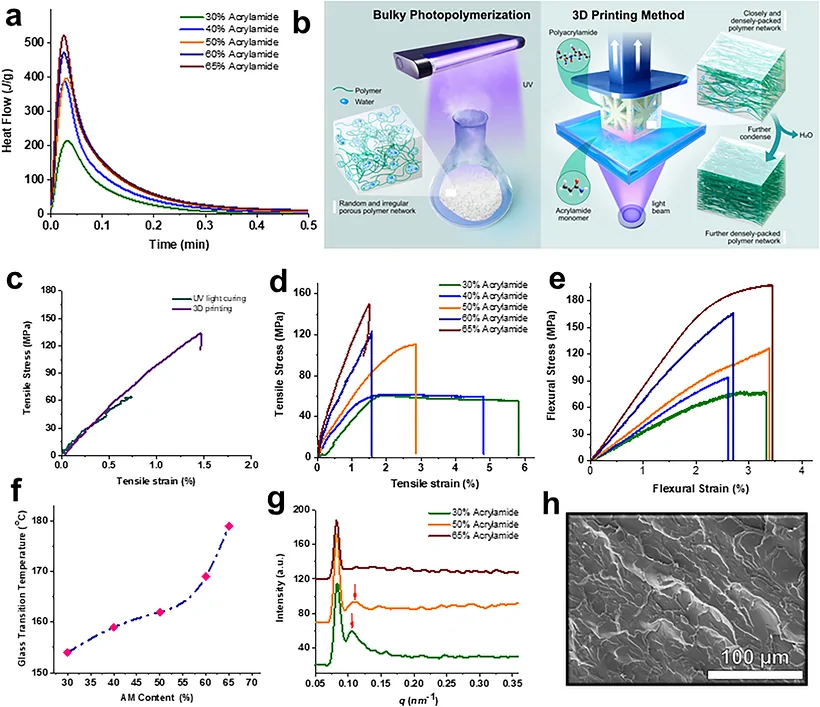
(a) Photo‐differential scanning calorimetry (Photo‐DSC) profiles showing accelerated exothermic reactions with higher acrylamide concentrations. (b) Schematic illustration of PAM networks formed via bulk photopolymerization (left) and 3D printing (right), highlighting how differing heat dissipation profiles during polymerization govern the resulting material quality. (c) Tensile stress‐strain curves of PAM fabricated by bulk photopolymerization and 3D printing. (d) Tensile and (e) flexural stress‐strain curves of PAM printed from inks with different acrylamide concentrations. (f) Effect of acrylamide concentration on the glass transition temperature (*T_g_*) of PAM. (g) Small‐Angle X‐ray Scattering (SAXS) profiles of PAM printed from inks containing 30, 50, and 65 wt.% acrylamide. (h) Cross‐section SEM image of PAM printed from ink with 65 wt.% acrylamide.

Additive manufacturing, particularly vat photopolymerization (VPP) methods such as stereolithography (SLA) and digital light processing (DLP), enables precise, layer‐by‐layer curing of polymers into complex geometries [27, 28, 29, 30, 31]. We found that this approach also allows PAM to be polymerized at an unusually high monomer concentration (65 wt.%) without problematic heat release. Because each thin layer contains only a small volume of reactive material, the generated heat can rapidly dissipate into the surroundings, preventing runaway exothermicity and yielding a uniform polymer network (Figure 2b).

To demonstrate this advantage, we used VPP to print PAM from a 65 wt.% acrylamide ink (with 0.5 wt.% LAP), followed by drying at 85°C until constant weight. The resulting PAM exhibited exceptional mechanical strength (Figure 1b). By contrast, bulk solution polymerization of the same formulation produced a highly exothermic reaction with rapid heat and steam release due to auto‐acceleration. The violent energy released vaporized water, expanded foam, and generated a porous, irregular network with poor mechanical properties (Figure 2b,c). Such uncontrollable exothermicity may explain why PAM has long been excluded from the ranks of high‐performance engineering polymers, despite its intrinsic potential.

Figure 2d,e and Figures S5 and S6 show the tensile, compressive, and flexural properties of PAM printed from inks containing 30%–65% acrylamide. Mechanical performance rose sharply with increasing monomer concentration: even PAM printed from 30% to 50% inks outperformed many conventional high‐performance engineering polymers, while concentrations above 60% yielded exceptional strength and modulus. These superior properties arise from two key factors. First, the exceptional mechanical performance of PAM originates from the highly entangled polymer network formed during the gel‐state polymerization process. As described in the experimental section, PAM was initially printed as a gel from an ink with a high content of AM without a chemical crosslinker, followed by gradual removal of free water to obtain the final dense polymeric material. Hence, the factors influencing polymer entanglement identified by Suo et al., can also help explain the degree of entanglement in the present system [32, 33]. As established by Suo et al., polymer chain entanglement is primarily governed by the water‐to‐acrylamide ratio (*W*) and the crosslinker‐to‐acrylamide ratio (*C*). Lower values of *W* and *C*—corresponding to higher monomer concentrations and minimal chemical crosslinking—favor the formation of densely entangled polymer chains, which effectively dissipate mechanical energy and enhance network toughness [32, 33]. In the present system, the crosslinker content is eliminated (*C* = 0), and *W* is remarkably lower than in hydrogel synthesis, conditions that strongly promote chain interpenetration and topological entanglement. Importantly, while the final PAM is glassy, it initially forms a soft, highly stretchable gel governed by these entanglement principles. Subsequent slow removal of water densifies this established network while largely preserving the initial topological entanglements. Also, this in situ polymerized PAM exhibits a high molecular weight (up to *M_v_* of 9.6 × 10^5^ g/mol, as determined by dilute solution viscometry in 0.5 M NaCl), which facilitates a high degree of topological entanglement. As a result, unusually robust and load‐bearing polymer architecture is expected to form, accounting for the observed high stiffness and strength. Second, the dense packing of PAM chains further contributes to the outstanding mechanical strength. The abundant polar amide groups along the polymer backbone promote strong intermolecular interactions, which enhance load transfer between adjacent chains [34, 35, 36]. Differential scanning calorimetry (DSC) revealed an increase in glass transition temperature (*T_g_*) with rising acrylamide concentration (Figure 2f), indicating that reduced chain mobility from tighter packing enhances mechanical performance [37]. Specifically, a significant increase in *T_g_* was observed, rising from 151°C at the typical 30 wt.% PAM concentration to 179°C at the upper limit of 65 wt.% PAM concentration. By contrast, PAM prepared from low‐concentration inks developed voids during drying, which markedly diminished tensile strength and modulus. This interpretation is supported by small‐angle X‐ray scattering (SAXS) and scanning electron microscopy (SEM).

SAXS patterns of PAM printed from 30% acrylamide showed a peak at *q* ≈ 0.094, corresponding to ∼67 nm voids, which shifted to *q* = 0.113 for 50% acrylamide, reflecting smaller domains with increasing monomer concentration (Figure 2g) [38]. Strikingly, PAM printed from 65% acrylamide exhibited no peak above *q* = 0.09, consistent with a nearly void‐free, fully dense structure, which is confirmed by the cross‐sectional SEM image (Figure 2h). At this highest concentration, the fracture surfaces also displayed multiple cracks, a hallmark of the material's high modulus. Together, SAXS and SEM establish that void formation is suppressed at high acrylamide loadings, enabling PAM to achieve exceptional mechanical strength.

### 2.3 Role of Water in Enhancing Polyacrylamide Mechanics

Mechanical testing revealed that water content critically governs the transition from a soft, gel‐like state to a high‐strength PAM polymer. As shown in Figure 3a, the PAM printed from 65% acrylamide ink exhibited gel‐like behavior with a tensile strength of only 0.28 MPa. Controlled drying progressively reduced the water content to 21.4, 14.3, and 11.6 wt.%, yielding tensile strengths of 29.5, 98.8, and 140.0 MPa, respectively, with the optimum strength achieved near 11.0 ± 0.6 wt.%. Further drying at elevated temperatures (180°C, vacuum) made the material brittle and weakened its performance. Notably, when the water content reached ∼11 wt.%, further heating at 85°C did not remove additional water, indicating a stoichiometrically bound fraction tightly associated with the polymer, similar to crystalline hydration water.

**FIGURE 3:**
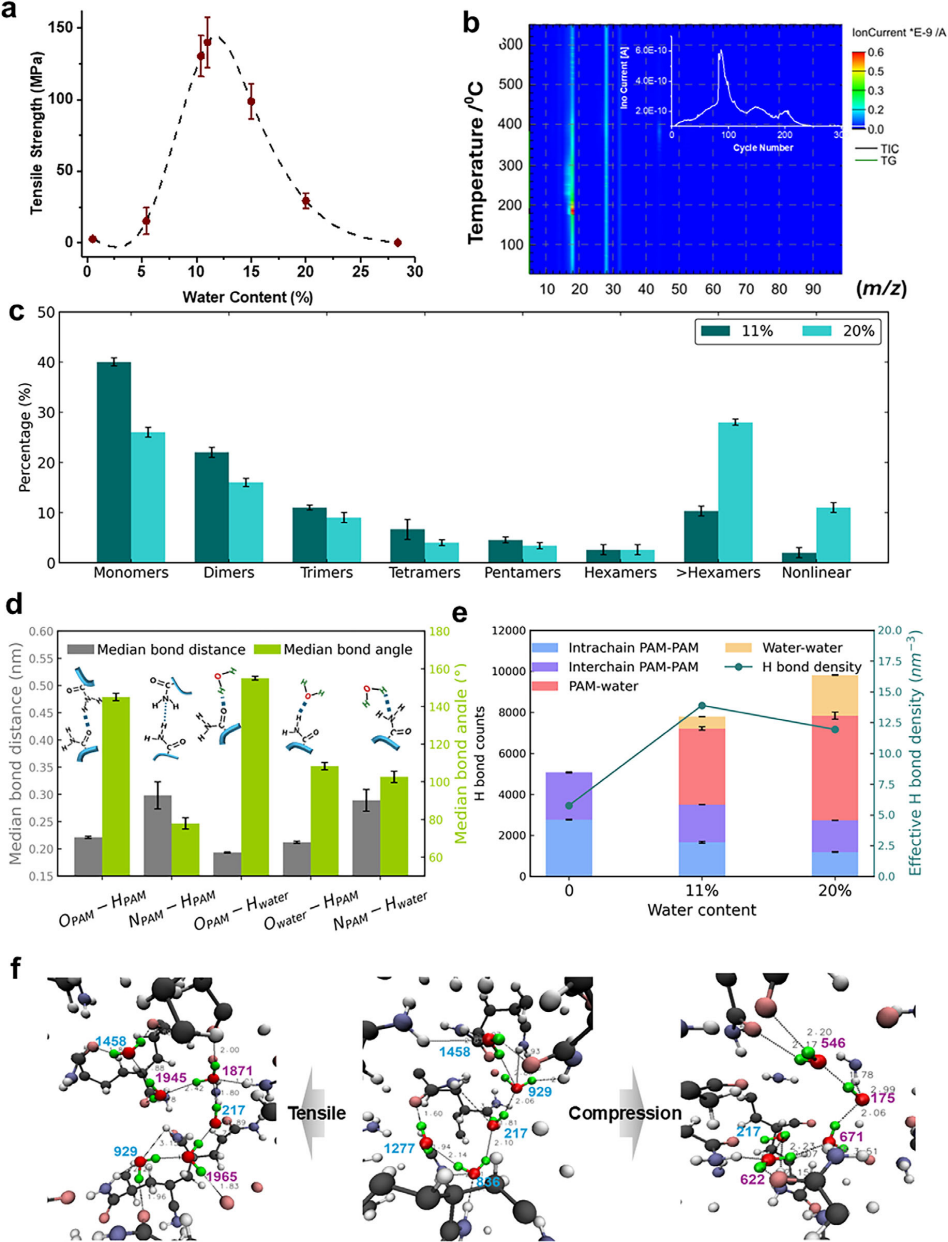
(a) Effect of water content on the tensile strength of PAM printed from 65% acrylamide ink. (b) Thermogravimetric analysis with mass spectrometry (TGA‐MS) contour plot showing temperature‐dependent evolution of gaseous species of PAM with bound water. (c) Histogram illustrating the percentages of water molecules in linear nanoclusters of varying sizes and in nonlinear nanoclusters (left to right), at 11% and 20% water content from MD simulations. (d) Median H‐bond distances (gray) and angles (green) at 11% water content for 2 types of PAM‐PAM H bonds (O_PAM_‐H_PAM_ and N_PAM_‐H_PAM_) and 3 types of water‐PAM H bonds (O_PAM_‐H_water_, O_water_‐H_PAM,_ and N_PAM_‐H_water_). Distances are calculated between the H atoms and the corresponding acceptor atoms, as illustrated in the schematics above. An ideal hydrogen bond adopts a linear conformation with a bond angle of 180°. More detailed H‐bond distances and angles are available in Figures S17 and S18. (e) The dependence of H‐bond counts and densities on water content. Bars (left axis) show the counts of different H‐bond types. The teal line (right axis) represents the density of effective load‐bearing hydrogen bonds (interchain PAM–PAM and PAM–water). (f) Snapshots illustrate the evolution of a linear water pentamer and the breaking and reforming of H bonds during tensile and 12 compressive deformations. After 1.5% tensile strain, the pentamer partially breaks up, with three molecules reorganizing into a hexamer together with three additional water molecules. Under 75% compressive strain, the pentamer fully breaks up. One of the water molecules forms a dimer. Numbers besides the water molecules indicate the unique residue identifiers of water molecules in the MD simulations, and all H bonds shorter than 0.35 nm are explicitly labeled. Additional snapshots of water nanoclusters under tensile and compressive loading are provided in Figures S21–S23.

To verify the presence of such strongly bound water, PAM samples printed from various acrylamide concentrations were dried at 85°C to remove free water and subsequently analyzed by thermogravimetric analysis (TGA). A pronounced weight‐loss event was observed at approximately 200°C (Figure S7), indicating the release of bound water that remained strongly associated with the PAM network after free‐water removal. This assignment was further confirmed by coupled TGA–mass spectrometry. As shown in the TGA–MS contour plot in Figure 3b and the inset, a sharp and intense *m/z* = 18 signal (red dashed line) appeared within a narrow temperature window, corresponding to the rapid release of water molecules. This water release at elevated temperature suggests that the retained water is not loosely trapped free water, but is strongly bound to the amide groups of PAM through hydrogen‐bonding interactions. Upon further heating, PAM degradation followed the previously reported pathway, with imidation of the amide side groups occurring at approximately 250°C, accompanied by imide formation and NH_3_ release (Figure S8; Figure 3b). At higher temperatures, around 400°C, polymer‐backbone scission became dominant [39]. To further investigate the chemical nature of these hydrogen‐bonding interactions, ATR Fourier‐transform infrared (FT–IR) spectroscopy was performed on 65% PAM samples before and after removal of free water (Figure S9). For PAM containing only bound water, the amide I band at around 1660 cm^−1^ shifts to a lower wavenumber because bound water forms specific C═O···H─O hydrogen bonds, weakening the carbonyl bond [40, 41, 42]. In contrast, PAM containing free water shows a much broader and stronger O─H stretching band at ∼3000–3600 cm^−1^, together with an enhanced band near ∼1640 cm^−1^ from H─O─H bending. These features suggest that excess water forms heterogeneous water–water hydrogen‐bonded clusters and partially screens PAM–PAM/PAM–bound‐water interactions [40, 43, 44, 45]. These results suggest that bound water participates in specific PAM–water–PAM hydrogen‐bonding bridges, whereas excess free water forms a bulk‐like hydration environment and screens the interchain amide interactions.

To uncover the molecular basis of the water‐induced strengthening of PAM, we conducted MD simulations at water contents of 0, 11, and 20 wt.%. All systems were equilibrated to match the experimental densities. Water clustering analysis of PAM containing 11% water, chosen based on experimental evidence that all water is bound, shows that water molecules are evenly distributed between the PAM chains, with 98% of water forming linear nanoclusters (Figure 3c; Figures S10–S12 and Table S4). 40% of water molecules exist as monomers, forming on average 3.9 H bonds per water molecule with surrounding PAM chains and fully saturating its bonding capacity (Tables S4 and S5). Despite the presence of larger nanoclusters, their linear geometry minimizes water‐water aggregation, thereby maximizing PAM–water interactions. Overall, each water molecule in the PAM‐water network forms an average of 3.1 out of 4 possible H bonds with PAM chains (Table S5), confirming that these water molecules are in a strong bonding state. Diffusion coefficient calculations further confirm this bonding state, as bound water in PAM displays significantly confined sub‐diffusive dynamics (Figures S13 and S14). Free Brownian motion emerged only above 500 K, when bound water was released, consistent with experimental observations (Figure 3b; Table S6).

Simulations reveal that the bound water‐induced strengthening of the PAM network arises from enhanced H‐bond strength, increased number of interchain H bonds and dynamic H bond network. First, H‐bond geometry analysis (Figure 3d; Figures S15–S18) reveals that PAM‐water H bonds are stronger than PAM‐PAM H bonds. The carbonyl oxygen of PAM interacting with the hydrogen atom of water (O_PAM_‐H_water_) forms the strongest H bonds, with the shortest median bond distance of 0.19 nm and a bond angle of 155° (Table S7), approaching the ideal values of hydrogen bonds [46, 47, 48]. The O_water_‐H_PAM_ and N_PAM_‐H_water_ pairs exhibit strengths comparable to the O_PAM_‐H_PAM_ and N_PAM_‐H_PAM_ pairs within PAM (Table S7). Moreover, Figure 3e indicates that compared with the fully dried PAM, bound water substitutes about 32% of the PAM‐PAM H bonds with a 73% increase in the number of the stronger PAM‐water H bonds, substantially increasing the total number of H bonds to strengthen the network.

Second, bound water in linear nanoclusters bridges adjacent chains, providing robust interchain reinforcement. Simulations show that PAM chains adopt a coiled conformation at the fully dried state (Figure S19), leading to about 55% of H bonds formed between H‐bonding sites within the same chains, as shown in Figure 3e. Consequently, under external loading, these PAM chains slide easily past one another, providing an explanation for the brittle behavior of dry PAM samples observed experimentally. In contrast, bound water extends PAM chain conformations and thereby reduces the fraction of intrachain PAM–PAM H bonds (Figure S19). Importantly, PAM‐water H bonds are predominantly interchain: 95.5% of water molecules reside in nanoclusters that bridge adjacent chains (Table S8). This extensive PAM–water network with a substantially higher density of effective load‐bearing H bonds (Figure 3e) resists chain sliding and enhances stress transfer, and thus the tensile strength. Furthermore, during tensile and compressive deformation (Figure 3f), water nanocluster distributions and H‐bond numbers remain largely unchanged, while individual H‐bonds and water nanoclusters continuously break and reform (Figures S20–S23). This dynamic yet robust H‐bond architecture enables sustained reinforcement, explaining the exceptional mechanical performance observed experimentally.

When water content further increases, larger and nonlinear water clusters form. Figure 3e shows that the presence of free water in larger water clusters expands the PAM matrix, reducing the density of effective H bonds despite the increase in PAM‐water H bonds (Figure S13). More importantly, diffusion coefficient calculations show significantly enhanced mobility of both water molecules and PAM chains, indicating these free water produces an overall plasticizing effect that significantly weakens the strength of PAM.

### 2.4 Self‐Extinguishing High‐Strength Polyacrylamides

Beyond their exceptional mechanical strength, PAM also exhibits an intrinsic self‐extinguishing capability, a property rarely achieved in synthetic polymer systems without additive flame retardants. The limiting oxygen index (LOI) of PAM reaches 46, far exceeding the critical range of 26–40 typically required for flame‐retardant polymers and polymer composites [49]. In standard UL‐94 vertical burning tests, poly(methyl methacrylate) (PMMA) was selected as the control sample because it is a representative polyacrylate possessing the typical polyacrylate backbone but without polar functional side groups capable of strong hydrogen‐bonding interactions. As shown in Figure 4a, PMMA ignites readily and sustains combustion, whereas PAM with bound water resisted ignition even after repeated ignition (Figure 4b). When ignition was sustained over a prolonged period, PAM consistently self‐extinguished within one second of flame removal, even after repeated attempts, highlighting its robust flame‐retardant properties (Figure S24).

**FIGURE 4:**
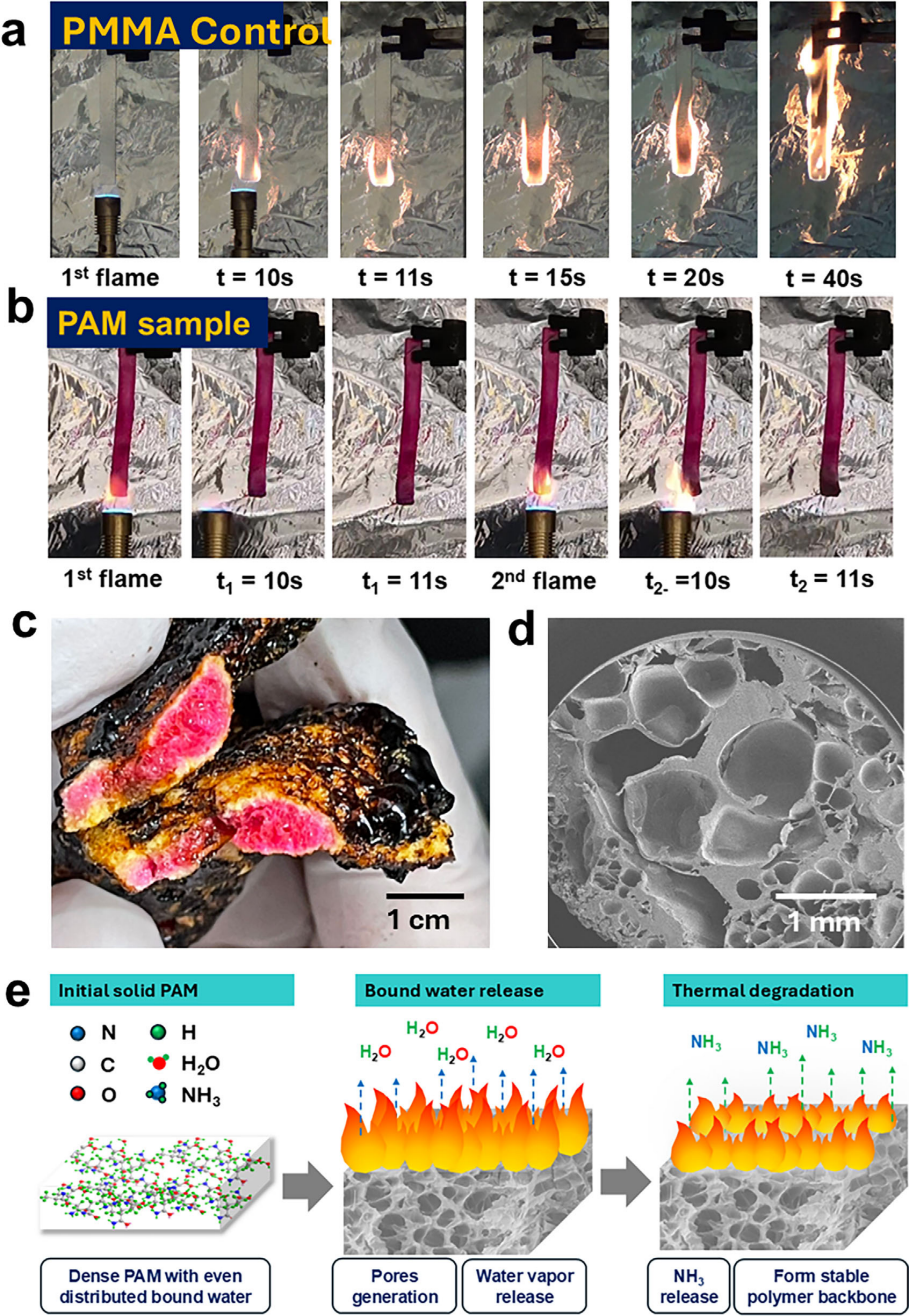
(a, b) Flame‐retardant testing of poly(methyl methacrylate) (PMMA, control) compared to PAM with bound water after ignition. (c) Cross‐sectional photograph and (d) scanning electron microscope (SEM) image of PAM specimen after flame‐retardant testing. (e) Proposed flame‐retardant mechanism of PAM with bound water, through the synergy of bound water release, NH_3_ generation, and imide formation.

Prolonged exposure to a high‐temperature flame (∼1,100°C) further revealed the mechanism underlying this unusual performance. During burning, PAM slowly developed a multilayered structure: a black residue layer, a porous middle layer, and a preserved inner core, as shown in Figure 4c. Remarkably, after 5 min of direct flame contact, the inner region of a 5 mm thick sample retained its original coloration from Rhodamine B (decomposition temperature ∼210°C), indicating that the interior remained below the decomposition temperature of Rhodamine B despite the extreme external heat. The in situ thermocouple measurements of the temperature inside and outside the PAM sample confirmed that the interior of a 2 mm thick sample remained below 200°C while the surface was directly exposed to flames exceeding 1,000°C (Figure S25).

The excellent self‐extinguishing capability of PAM is attributed to the synergistic effects of bound water and the intrinsic thermal chemistry of the amide‐containing polymer backbone. Upon heating to approximately 200°C, bound water is rapidly released from the PAM matrix, generating a large amount of water vapor that can quench the flame and form a highly porous internal structure (Figure 4d). To further clarify the contribution of bound water to the flame‐retardant behavior, UL‐94 vertical burning tests were also conducted using anhydrous PAM samples. Interestingly, anhydrous PAM also exhibited excellent self‐extinguishing behavior, although the foaming and pore‐forming process was much less pronounced than that observed in PAM with bound water. As shown in Figure 4d, PAM with bound water formed a highly porous residue with abundant micro‐ to meso‐pores after combustion, whereas anhydrous PAM produced only a limited number of large macropores (Figure S26). This difference can be attributed to the rapid release of bound water during combustion, which acts as an internal blowing agent and generates numerous micro‐ and meso‐scale pores throughout the material. In contrast, anhydrous PAM lacks sufficient internal water to produce such a foaming effect, and the limited macropore formation is mainly caused by gaseous products released during direct polymer decomposition, such as NH_3_ evolution during thermal degradation (Figure S8).

TG–MS analysis further revealed the evolution of gaseous species, including NH_3_ and NH_2_• radicals, above 200°C (Figure 3b), consistent with the proposed thermal degradation pathway of PAM (Figure S8). These degradation processes are accompanied by the formation of imide structures, as confirmed by FT–IR analysis of the burnt residue (Figure S27) [50, 51]. Importantly, imide formation helps to stabilize the polymer backbone and enhances its thermal resistance [52, 53]. This interpretation is further supported by the relatively narrow CO_2_ evolution window observed at 350°C–420°C in TG–MS analysis (Figure 3b), suggesting delayed and more confined oxidative decomposition of the stabilized polymer structure. Based on these results, the exceptional self‐extinguishing behavior of PAM with bound water can be understood as a synergistic mechanism (Figure 4e): (i) the rapid release of bound water provides a cooling effect and generates an expanded porous structure that acts as a thermal barrier; (ii) thermal degradation of amide groups releases NH_3_, which further suppresses combustion by diluting combustible gases and interfering with flame propagation; and (iii) progressive imide formation stabilizes the polymer backbone, leading to a thermally more stable protective residue. Together, these effects account for the outstanding flame‐retardant performance and rapid self‐extinguishing behavior of PAM.

## 3 Conclusion

In summary, this work demonstrates that the role of water in synthetic polymers can be fundamentally redefined through controlled polymer–water interactions. We show that a critical fraction of bound water (∼11 wt.%) in PAM forms a uniform and enhanced hydrogen‐bond network with amide groups along the polymer backbone. Molecular dynamics simulations reveal that the bound water exists predominantly as monomers and linear nanoclusters that act as effective physical interchain crosslinkers, increasing the overall hydrogen‐bond density and maintaining network integrity under both tensile and compressive deformation. Experimental results directly validate these predictions, with the resulting PAM exhibiting exceptional stiffness, strength, and load‐bearing capability, including a tensile modulus exceeding 9 GPa and a tensile strength above 140 MPa. Furthermore, the material achieves an intrinsic self‐extinguishing capability (LOI 46) through the synergistic release of bound water and ammonia and the formation of a stable, protective polymeric backbone without external additives. These findings elucidate the molecular‐level origin of water‐enabled reinforcement in a synthetic polymer system and establish bound water as a quantifiable, load‐bearing component rather than a plasticizing impurity. This study redefines the role of water in high‐performance polymers, demonstrating that water can actively enhance polymer strength, offering a new design strategy for high‐performance synthetic polymers.

## Author Contributions

**Yi Ting Chong**: methodology, data curation. **Junhua Kong**: validation, data curation. **Sirawit Pruksawan**: methodology, data curation, validation, writing – original draft. **Xian Jun Loh**: writing – review and editing, supervision, conceptualization. **FuKe Wang**: conceptualization, methodology, supervision, project administration, writing – original draft, writing – review and editing.

## Funding

This work was supported by the SERC Central Research Fund (TIMR211001bSERCRF) and the MTC Individual Research Grants (M23M6c0110) from A*STAR of Singapore, A*STAR Career Development Fund (C233312015, C233312002) from the A*STAR of Singapore.

## Conflicts of Interest

The authors declare no conflicts of interest.

## Supporting information




**Supporting File**: advs76537‐sup‐0001‐SuppMat.docx.

## Data Availability

The data that supports the findings of this study are available in the supplementary material of this article.
