# Supplementary material for: Bound Water as a Reinforcing Element for Ultra‐Strong Polyacrylamide
Source: Adv Sci (Weinh). 2026 Jul 13;13(58):e76537. doi: 10.1002/advs.76537 (PMC13360102; doi:10.1002/advs.76537)
Supplement: Supplementary file 1 [file ADVS-13-e76537-s001.docx]

Supporting Information

**Bound Water as a Reinforcing Element for Ultra-Strong Polyacrylamide**

Sirawit Pruksawan, Nannan Li, Zehuang Lin, Jianwei Zheng, Terence Jun En Loh, Yi Ting Chong, Junhua Kong, Xunan Hou, Xian Jun Loh, Chaobin He, FuKe Wang*

**Experimental Details**

1. Material preparation and characterization

1-1. Experimental Materials

Acrylamide (AM) (≥ 99% purity) was obtained from Sigma-Aldrich (Singapore) and used as received. Additionally, lithium phenyl-2,4,6-trimethylbenzoylphosphinate (LAP) was acquired from TCI Chemical (Singapore) and used in its original state.

**1-2. Preparation of polyacrylamide samples**

The polyacrylamide (PAM) precursor was prepared as follows: A specified quantity of acrylamide monomer (30, 40, 50, 60, and 65 *wt.*%) was dissolved in deionized water through ultrasonic treatment at 60°C until complete dissolution. Subsequently, LAP photoinitiator (0.5 *wt*.%) was added to the dispersion and sonicated to ensure uniform dissolution. All percentages mentioned in this paper refer to mass percentages. The precursors obtained were 3D printed using an Anycubic Photon M3 printer (Shenzhen Anycubic Technology Co., Ltd., China). A model in STL (Stereolithography) format was imported into the Anycubic Photon Workshop 3D Slicer software to create the slicing file. Each printing layer was set at a thickness of 0.05 mm with a light intensity of 70%. The exposure time for each layer ranged from 3 to 30 seconds. After importing the slicing file, the prepared precursor was poured into the printer’s vat, and the printing process occurred layer by layer. Subsequently, the printed specimens were rinsed in deionized water and underwent post-curing in a UV oven to produce the samples. The obtained samples in the wet state were stored in the refrigerator until further use.

For comparison, bulk photopolymerization of PAM was also performed using a reduced amount of ink in a glass vial. The same ink formulation used for 3D printing was transferred into an open vial (≤5 mL) and irradiated in a UV oven equipped with four 15 W UV lamps. To prevent explosive photopolymerization, the reaction was initiated by exposing the vial to UV light for 5 s, followed by cooling to room temperature before re-initiating UV irradiation until polymerization was complete.

To produce dried PAM samples, the wet samples were covered with glass slides to maintain their shape and dehydrated in an oven. The oven temperature was gradually increased from room temperature to 85 °C and maintained at this temperature for more than 48 hours until no further weight change occurred. Additionally, vacuum oven drying was performed at 85 °C under vacuum conditions. The resulting polymer samples were stored in a dry cabinet for subsequent testing and characterization.

**1-3. Characterization of polyacrylamide samples**

**Thermal analysis**

Photo-differential scanning calorimetry (Photo-DSC) was conducted using a Q100 apparatus (TA Instruments, Germany) equipped with a photo calorimeter accessory (Novacure 2100, EXFO Photonic Solutions Inc) under a nitrogen flow at 50 ml/min. Approximately 5 mg of PAM precursor (in the uncured state) was placed in a hermetic pan, with an empty hermetic pan serving as the reference material. The heat of reaction (*ΔH_r_*) for each sample was determined by integrating the area under the exothermic heat release curve using TA Universal Analysis Software (TA Instruments, Germany).

Differential scanning calorimetry (DSC) was carried out using a Q100 apparatus (TA Instruments, Germany) with a nitrogen flow rate of 50 ml/min. Dehydrated PAM samples, approximately 5 mg each, were sealed in a hermetic pan, while an empty hermetic pan served as the reference material. The samples were subjected to two heating cycles, from 25 to 200 °C, at a rate of 20 °C/min. Glass transition temperatures (*T_gs_*) were determined during the second heating scan of the samples.

Thermogravimetric analysis (TGA) was performed in a nitrogen atmosphere using a Q500 analyzer (TA Instruments, Germany). Approximately 20 mg of dehydrated polymer samples were loaded into aluminum pans, with an empty aluminum pan serving as the reference material. The temperature was gradually increased from around 20 to 500 °C at a heating rate of 20 °C/min. Thermogravimetric analysis/mass spectrometry (TGA/MS) was conducted using TG 209 F1 Libra (NETZSCH, Germany) under similar experimental conditions as TGA, except for a lower heating rate of 5 °C/min.

**Mechanical analysis**

Mechanical tensile, flexural, and compressive tests were conducted employing an Instron 5569 double-column universal testing machine (Instron Corporation, United States). Tensile tests utilized a 45-kN load cell for PAM samples. Nominal tensile strain was calculated by normalizing the displacement with the initial gauge length of the specimen. PAM samples in a tensile bar shape were clamped with pneumatic grips and extended at strain rates of 4 mm/minute until fracture occurred. Tensile strength was calculated by dividing the maximum load by the original cross-sectional area of the test specimen. The tensile modulus was obtained from the linear segment of the measured tensile stress-strain curves. The flexural test was conducted on PAM samples using a 45-kN load cell, employing rectangular bar-shaped samples. These samples were placed between two support pins set 48 cm apart and subjected to bending by applying force at the center, with a strain rate of 1.2 mm/minute. The flexural strength was determined by dividing the maximum load by the product of the span length and the specimen's width, while the flexural modulus was derived from the linear section of the initial flexural stress-strain curves. The compressive test was conducted using a 45-kN load cell on cylindrical samples with a diameter of 7.5 mm and a height of 15 mm, at a rate of 1 mm/minute. The compressive yield strength was calculated by dividing the load at the yield point by the cross-sectional area of the sample and the compressive modulus was determined from the linear section of the initial compressive stress-strain curves. To ensure the reproducibility of these mechanical tests, a minimum of three samples were tested under each condition, and the collected data were averaged.

To demonstrate the mechanical strength of the prepared PAM, a plate (20 cm × 10 cm × 5 mm) was 3D printed using an LCD 3D printer. The printed plate was dried in a vacuum oven at 85 °C for approximately 2 days to remove free water. The dried plate was then fixed to a chuck, and a student weighing 60 kg stood on it. No visible bending or fracture was observed, indicating excellent load-bearing capacity.

To demonstrate stability in water, the PAM sample was coated with a uniform silicone layer (~1 mm thick) using SYLGARD 184. A tripod-shaped PAM sample (0.6 g) was immersed in water, and a 6 kg metal block was placed on top. No deformation or structural failure was observed after 5 days of immersion, confirming the strong supporting ability of PAM under wet conditions.

**Morphological and structural Analysis**

Small-angle X-ray scattering (SAXS) was employed to characterize the structural characteristics of dehydrated PAM samples. The experiments were conducted on a Xenocs Xeuss 2.0 instrument using ultralow divergence Cu Kα radiation at 50 kV and 60 mA, with a total exposure time of 300 seconds. Transmittance was measured as *T* = *I*/*IB*, where *I* and *IB* represent the direct-beam intensity with and without a specimen, respectively, collected in a 5 by 5-pixel area at the center of the pattern. 2D SAXS scattering images were collected from the instrument. 1D scattering intensity profiles were obtained by integrating in equatorial and meridional directions. The SAXS intensities were normalized by sample thickness and transmissions.

Field-emission scanning electron microscopy (FE-SEM) was employed for the characterization of the cross-section microstructure of dried PAM samples. Generally, the dried PAM tensile bar samples were first immersed in liquid nitrogen for approximately 10–15 min and subsequently fractured to expose the cross-sectional surfaces. The fractured samples were then dried under vacuum, mounted onto aluminum stubs, and sputter-coated with a thin gold layer for 25 s using a JEOL JFC-1600 Auto Fine Coater prior to SEM observation.

**Physicochemical property analysis**

The water content of PAMs was determined using samples with a circular shape and a thickness of 0.2 cm. After removing surface moisture from the samples by wiping them with paper, the initial weight (*W_i_*) was measured. To prepare the anhydrous PAM samples, the small pieces of PAM samples was first dried as normal and then subsequent dried in a vacuum oven (~ 10 mbar) at 180 °C for at least 2 hours. Following this process, the dried weight (*W_d_*) was measured, and the water content of the samples was calculated using the equation: Water content (%) = (*W_i_* - *W_d_*) / *W_i_* × 100. The density of polymer samples was measured with a micromeritics AccuPyc II 1340 gas pycnometer.

**Chemical Analysis**

Fourier-transform infrared (FTIR) spectroscopy was performed using a PerkinElmer Spectrum 2000 FTIR spectrometer (PerkinElmer, Waltham, MA, USA) operated in attenuated total reflectance (ATR) mode. Spectra were recorded in the range of 4000–400 cm⁻¹ by accumulating 64 scans per spectrum with a resolution of 4 cm⁻¹.

**Molecular Weight Estimation**

The viscosity-average molecular weight (*M_v_*) of the 65 wt. % PAM specimen (dry state) was determined via dilute solution viscometry using a Cannon-Fenske viscometer at 25 °C. To suppress polyelectrolyte expansion, the polymer was dissolved in a 0.5 M NaCl aqueous solution. At a dilution of 0.02 wt.% (*c* = 0.0002 g/mL), the intrinsic viscosity (*[η]*) was approximated by the reduced viscosity (*η_sp_/c*), yielding a value of 348.5 mL/g. The *M_v_* was subsequently calculated using the Mark-Houwink-Sakurada relationship (*[η]* = *K · M_v_^a^*) ^[1]^ with established empirical constants for PAM in salt solution (*K* = 0.012 mL/g and *a* = 0.75) ^[2]^, resulting in an estimated *M_v_* of 9.6 × 10^5^ g/mol. For comparison, the *M_v_* of the specimen representing the lower limit of investigated monomer concentration (30 wt.% PAM) was determined to be 4.2 × 10^5^ g/mol.

**1-4. Flame Retardant analysis**

Vertical burning tests were conducted according to UL-94 using 3D-printed samples (127 × 12.7 × 1.6 mm). Control samples (polyHEMA) burned continuously after ignition, whereas PAM samples self-extinguished immediately upon flame removal. Hence, a second ignition was applied to confirm the excellent flame-retardant behavior of the PAM samples. Limiting oxygen index (LOI) was measured on 3D-printed samples (80 × 10 × 1.6 mm) following ASTM D2863. The minimum oxygen concentration sustaining combustion was recorded, demonstrating the improved flame retardancy of the PAM samples compared with the control.

**1-5. Method for estimating heat generation during single-layer curing**

Despite a substantial heat reaction of around 528 J/g for PAM with a 65% concentration, the thin polymerization layer (typically 50 µm thick) in 3D printing produces only about 1.7 J of heat for a 10 mm × 10 mm film layer, easily dissipated by the surrounding solution.

**2. Simulation Methods and Supporting Data**

2-1. **Molecular Dynamics (MD) simulation protocol**

All-atom molecular dynamics (MD) simulations were performed using the GROMACS 2021 software package^[3]^. We adopted the bonded and nonbonded potential parameters from CHARMM General Force Field for PAM and the TIP3P model for water ^[4]^. The cutoff distance for short-range electrostatic and van der Waals interactions was 1.2 nm. For long-range electrostatics, the particle mesh Ewald method has been implemented. To control the temperature, the v-rescale thermostat was used with a coupling constant of 0.1 ps. For NPT simulations, the Parrinello−Rahman pressure coupling with coupling constant of 5.0 ps was used.

To prepare the initial simulation systems, a single polyacrylamide (PAM) chain with 100 repeat units was first equilibrated in water using the equilibration process as listed below, where consecutive NVT and NPT steps give rise to efficient packing of the polymer chain in water. The equilibrated single PAM chain with appropriate numbers of water molecules chosen from its first solvation shell were then used to generate the initial simulations systems of 40 PAM chains with water contents 11% and 20%, which correspond to 2,000 and 4,000 water molecules, respectively. We also prepared a system of 40 PAM chains with no water molecules.

***Equilibration steps for preparing initial systems polyacrylamide (PAM) at various water content.***

| **Steps** | **Ensemble** | **Temperature (K)** | **Pressure (bar)** | **Time (ns)** |
| --- | --- | --- | --- | --- |
| 1 | NVT | 298 | N.A. | 5 |
| 2 | NPT | 298 | 1 | 5 |
| 3 | NVT | 298 | N.A. | 5 |
| 4 | NPT | 298 | 500 | 5 |
| 5 | NVT | 298 | N.A. | 5 |
| 6 | NPT | 298 | 3000 | 5 |
| 7 | NVT | 298 | N.A. | 5 |
| 8 | NPT | 298 | 500 | 5 |
| 9 | NVT | 298 | N.A. | 5 |
| 10 | NPT | 298 | 1 | 5 |
| 11 | NVT | 298 | N.A. | 5 |
| 12 | NVT | 800 | N.A. | 50 |
| 13 | NVT | 600 | N.A. | 50 |
| 14 | NVT | 500 | N.A. | 50 |
| 15 | NVT | 400 | N.A. | 50 |
| 16 | NVT | 298 | N.A. | 50 |
| 17 | NPT | 298 | 1000 | 5 |
| 18 | NVT | 298 | N.A. | 50 |

The three initial systems (0%, 11% and 20% water content) were first equilibrated by consecutive NVT and NPT steps as shown by steps 1 to 11 as listed above at room temperature to maximize the interactions between water molecules and PAM. At step 11, the system densities were close to their respective final target densities. We then applied long NVT simulations at elevated temperatures (steps 12 to 16) to further equilibrate the system. Lastly, we applied NPT simulations at 1,000 bar (step 17) to achieve the target experimental densities of 1.33g/cm^3^ at 0% and 11%, and 1.06 g/cm^3^ at 20%, followed by production runs in NVT for 50 ns. Such an equilibration process is to ensure that water molecules can effectively diffuse and interact within the PAM matrix, resulting in their even and adequate distribution.

Tensile and compression were performed using the deform option by specifying a constant strain rate in the z direction. For tensile deformation, we tested two different strain rates 2×10^-5^ and 2×10^-6^ nm/ps, and for compression deformation, we used 10^-3^ and 5×10^-4^ nm/ps. The effects on hydrogen (H) bonding in the 11% system are independent of the strain rates. Semi-isotropic pressure coupling was also adopted where the compressibility in the *z* direction was set to zero to allow deformation in the *z* direction to occur.

2-2. **Computational methods for analyzing water molecule distributions in PAM**

To gain a deeper understanding of the spatial distribution of water molecules in the PAM matrix, we applied a clustering analysis algorithm based on the 3D positions of water molecules from MD simulations. In this approach, a neighboring water molecule is considered part of a cluster if it is within an O_water_-H_water_ distance of 0.2 nm. This criterion was applied iteratively: for each water molecule identified as part of the cluster, its neighboring molecules within the 0.2 nm threshold are also included in the same cluster. This process continues until no additional water molecules within the specified O_water_-H_water_ distance are found. The resulting group of water molecules is then classified as a single molecular water nanocluster. The cutoff distance of 0.2nm was chosen because the average O_water_-H_water_ distance in water is 0.19nm from our MD simulations and the literature ^[5]^.

To understand the extent of water-water clustering and nonlinearity in each molecular water nanocluster, it is intractable to visually inspect the thousands of water nanoclusters identified in MD simulations. Here, we take a more systematic approach by converting each water nanocluster into a graph where the position of the oxygen atom is taken as the vertex. If the O_water_-O_water_ distance between the two water molecules is smaller than 0.3 nm, we draw an edge to connect the two vertices that represent these two water molecules. This O_water_-O_water_ cutoff distance was chosen because the average O_water_-O_water_ distance in water is 0.29 nm from our MD simulations and the literature ^[6]^.

In graph theory, a cycle is a path that starts and ends at the same vertex, with no repeated edges or vertices (except for the starting and ending vertex). In linearly distributed water nanoclusters, no cycles are present and water-water interactions are minimized. In contrast, in a nonlinear clustered group of water molecules, cycles can be identified, suggesting water-water interactions are increased and their contact with the surrounding polymer matrix is reduced. The snapshots were prepared using VMD.^7^

2-3. **Radial distribution functions (RDFs)**

To systematically compare the H bonding interactions, we plot the radial distribution functions (RDFs) between the donor and acceptor pairs. Comparisons of RDFs at 0%, 11% and 20% are shown in Figure S3. An RDF plot describes how the density of particles varies as a function of distance r from a reference particle. In an RDF plot, peaks indicate preferred distances where particles are more likely to be found. The position of the peak corresponds to the most probable distance between particles, reflecting specific interactions, such as H bonds in this case. The height of the peak indicates the strength or frequency of interactions at that distance, and higher peaks mean stronger or more frequent interactions.

2-4. **H bond analysis**

Beyond RDF, we analyzed the distances and angles of PAM–PAM and PAM–water H bonds formed between all donor–acceptor pairs at 0%, 11%, and 20% water content, as indicators of H bond strength. In measuring of the number of H bonds per monomer water molecule, a cutoff distance of 0.35nm was chosen to identify PAM-water H bonds based on the first minima of the PAM-water RDFs.

2-5. **H bond analysis during tensile stretching and compression**

Tensile deformation up to 1.5% strain was performed on the 11% samples in MD simulations. We have also performed compression up to 75% on the 11% samples and examine the number of H bonds during either tensile or compression deformation. The density increased slightly from 1.33g/cm^3^ to 1.35g/cm^3^ during the compression.

2-6. **Diffusion coefficients of water molecules and PAM chains**

Diffusion coefficients of water molecules at 11% and 20% water content at temperatures between 298K and 800K. Diffusion coefficients were calculated using *gmx msd* and fitting the MSD vs. time curves according to $MSD\propto t^{\alpha}$. If $\alpha=1$, the Brownian diffusion coefficient, *D*, is calculated as $MSD=6Dt$. If $\alpha<1$, the water molecules exhibit subdiffusive behavior.

Brownian diffusion was observed for both 11% and 20% for temperatures at 500K and above, providing support for the release of bound water at 180°C observed experimentally. At temperatures of 400K and below, sub-diffusive behavior was observed, where $MSD\propto t^{\alpha}$, and $\alpha<1$. At all temperatures, water molecules at 20% exhibit more mobility than those at 11% water content.

**Supporting Figures**

Figure S1. Ashby plot showing the position of our 65% PAM relative to other engineering polymers, including polyimide (PI), polyether ether ketone (PEEK), polyethylene (PE), and polyamide-imide (PAI), as well as composites, wood, and metals, in terms of tensile strength and modulus. Reference: M. F. Ashby, in *Materials Selection in Mechanical Design,* G. E. Dieter, Ed. (Butterworth-Heinemann, Oxford, United Kingdom, 1997), pp. 57-96.

Figure S2. Tensile stress–strain curves of polyacrylamide (PAM) hydrogels with different water contents.

Figure S3. Stability test of silicone-coated PAM with bound water supporting a 6 kg load in a water bath for 5 days. The material exhibited minimal height change and no noticeable swelling or mechanical degradation, with a negligible weight gain of 0.84% recorded after 5 days.

Figure S4. Measured heat of reaction (*ΔH_r_*) of polyacrylamide (PAM) precursors with varying acrylamide concentrations obtained from photo-differential scanning calorimetry (photo-DSC).

Figure S5. (A) Compression behavior of PAM containing 65 wt% acrylamide, showing no fracture even at the maximum load limit of the tensile tester. (B) Full-range compressive stress-strain curves of polyacrylamides (PAMs) with varying acrylamide concentrations.

Figure S6. (a) Tensile strengths, compressive yield strengths, and flexural strengths; (b) Tensile modulus, compressive modulus, and flexural modulus of polyacrylamides (PAMs) plotted as a function of acrylamide concentrations.

Figure S7. Thermogravimetric analysis (TGA) profiles of dried polyacrylamide (PAM) printed from ink at different acrylamide concentrations.

Figure S8. Thermal degradation pathway of PAM through imidiation.

Figure S9. ATR-Fourier Transform Infrared Spectroscopy (FTIR) spectra of PAM containing exclusively bound water against PAM sample coexisting bound and free water.

Figure S10. Snapshots of single water molecules from 11% water content. The distances between the oxygen and hydrogen atoms of water with surrounding PAM atoms are labelled in the unit of Å. The color code is as follows: red for the oxygen atoms of water, green for the hydrogen atoms of water, pink for the carbonyl oxygen atoms of PAM, iceblue for the nitrogen atoms of PAM, gray for the carbon atoms of PAM, and white for the hydrogen atoms of PAM.

Figure S11. Snapshots of (a) and (b) dimers, (c) and (d) linear trimers, and (e) and (f) linear tetramers from 11% water content. The distances atoms are labelled in the unit of Å. The color code is as follows: red for the oxygen atoms of water, green for the hydrogen atoms of water, pink for the carbonyl oxygen atoms of PAM, blue for the nitrogen atoms of PAM, gray for the carbon atoms of PAM, and white for the hydrogen atoms of PAM.

**Figure S12.** Snapshots of (a) a linear pentamer, (b) a linear hexamer, (c) a linear heptamer, (d) a linear octamer (8 water molecules), (e) a linear nonamer (9 water molecules), and (f) a linear decamer (10 water molecules) from 11% water content. The distances between the oxygen and 5 atoms of water molecules are labelled in the unit of Å. The color code is as follows: red for the oxygen atoms of water and green for the hydrogen atoms of water. The surrounding PAM atoms are displayed as transparent to provide a clearer visualization of the water molecule conformations within the nanoclusters.

**Figure S13.** (a) Averaged mean squared displacement (MSD) of all water molecules at 11% and 20% vs. time. α is defined as the exponent of $MSD\propto t^{\alpha}$, where α = 1 is normal Brownian diffusion and α <1 is restricted diffusion in the sub-diffusive regime. (b) Averaged MSD of monomers, a 15-mer, and all water molecules irrespective of the size of molecular water nanoclusters they belong at 11% water content vs. time.

**Figure S14.** (a) Averaged mean squared displacement (MSD) of all PAM chains at 0%, 11% and 20% vs. time. (b) Averaged MSD at 5ns of all PAM chains vs. water content.

Figure S15. RDF plots of PAM-water H bond interactions between (a) O_PAM_-H_water_, (b) N_PAM_-H_water_, (c) O_water_-H_PAM_. (d) RDF plots of water-water H bond interactions between O_water_-H_water_. (e) and (f): RDF plots of PAM-PAM H bonds between O_PAM_-H_PAM_ and N_PAM_-H_PAM_. All RDF plots were obtained at 11% water content.

Figure S16. RDF plots at various water contents of PAM-water H bond interactions between (a) O_PAM_-H_water_, (b) N_PAM_-H_water_, (c) O_water_-H_PAM_. (d) RDF plots of water-water H bond interactions between O_water_-H_water_. (e) and (f): RDF plots of PAM-PAM H bonds between O_PAM_-H_PAM_ and N_PAM_-H_PAM_.

Figure S17. H⋯Acceptor distance distributions of PAM-PAM H bonds at (a) 0%, (c) 11% and (e) 20% water contents. Angle distributions of PAM-PAM H bonds at (b) 0%, (d) 11% and (f) 20% water contents. 180° represents an ideal and linear H bond.

Figure S18. H⋯Acceptor distance distributions of water-PAM H bonds at (a) 11% and (c) 20% water contents. Angle distributions of water-PAM H bonds at (b) 11% and (d) 20% water contents. 180° represents an ideal and linear H bond.

**Figure S19.** Snapshots of PAM chains from MD simulations at (a) 0%, (b) and (c) 11% and (d) 20% water contents.

Figure S20. The number of intrachain and interchain PAM-PAM H bonds, PAM-water H bonds and water-water H bonds of the 11% PAM-water network up to (a) 1.5% tensile strain and (b) 75% compression strain. The red bars represent the densities of effective load-bearing H bonds (interchain PAM-PAM and PAM-water H bonds) up to 1.5% tensile and 75% compression strain.

**Figure S21.** Snapshots of a monomer water molecule from the MD simulations at 11% water content during tensile and compression deformation. After 1.5% tensile deformation, it forms a trimer with two other water molecules. At 75% compression strain, it forms a tetramer with three other water molecules. The numbers next to the water molecules are the unique residue numbers assigned to each water molecule in MD simulations. The color code is as follows: red for the oxygen atoms of water, green for the hydrogen atoms of water, pink for the carbonyl oxygen atoms of PAM, iceblue for the nitrogen atoms of PAM, gray for the carbon atoms of PAM, and white for the hydrogen atoms of PAM. New water molecules that come near to the original PAM segments to form new hydrogen bond interactions are colored cyan.

**Figure S22.** Snapshots of a dimer water nanocluster from the MD simulations at 11% water content during tensile and compression deformation. After 1.5% tensile deformation, it breaks up and one of the molecules forms a tetramer with three other water molecules. At 75% compression strain, it breaks up and one of the molecules forms a dimer with another water molecule. The numbers next to the water molecules are the unique residue numbers assigned to each water molecule in MD simulations. The color code is as follows: red for the oxygen atoms of water, green for the hydrogen atoms of water, pink for the carbonyl oxygen atoms of PAM, iceblue for the nitrogen atoms of PAM, gray for the carbon atoms of PAM, and white for the hydrogen atoms of PAM. New water molecules that come near to the original PAM segments to form new hydrogen bond interactions are colored cyan.

**Figure S23.** Snapshots of an 11-mer water nanocluster from the MD simulations at 11% water content during tensile and compression deformation. After 1.5% tensile deformation, it breaks up and four of the molecules form a heptamer with three other water molecules. At 75% compression strain, it breaks up and one of the molecules forms a pentamer with four other water molecules. The numbers next to the water molecules are the unique residue numbers assigned to each water molecule in MD simulations. The color code is as follows: red for the oxygen atoms of water, green for the hydrogen atoms of water, pink for the carbonyl oxygen atoms of PAM, iceblue for the nitrogen atoms of PAM, gray for the carbon atoms of PAM, and white for the hydrogen atoms of PAM. New water molecules that come near to the original PAM segments to form new hydrogen bond interactions are colored cyan.

Figure S24. Forces ignition of the PAM with bound water and its self-extinguish ability

Figure S25. Temperature evolution inside and outside the PAM plate (2 mm thickness). (A) Photo of the experimental setup. (B) Temperature profiles recorded by thermocouples embedded within (red) and positioned outside (blue) the PAM plate. (C) Schematic representation of the experimental configuration.

Figure S26. Comparison of the cross-section SEM images of the burnt PAM samples with bound water (left) and without bound water (right).

Figure S27. FT-IR spectra of the burnt reside

Supporting Tables

Table S1. Tensile strength of 65% PAM (Type V specimen) measured and certified by TUV SUD PSB Pte Ltd under ASTM D638-22. Test report number 7191324949-MEC24-AY.

| **Material: 65% PAM** | **Tensile strength (MPa)** |
| --- | --- |
| Specimen #1 | 136.8 |
| Specimen #2 | 148.0 |
| Specimen #3 | 145.2 |
| Specimen #4 | 145.6 |
| Specimen #5 | 123.9 |
| **Average** | **139.9** |

Table S2. Tensile strength of 65% PAM (Type IV specimen) measured and certified by TUV SUD PSB Pte Ltd under ASTM D638-22. Test report number 7191324949-MEC24-AY.

| **Material: 65% PAM** | **Tensile strength (MPa)** |
| --- | --- |
| Specimen #1 | 105.2 |
| Specimen #2 | 137.0 |
| Specimen #3 | 129.8 |
| Specimen #4 | 104.1 |
| Specimen #5 | 172.4 |
| **Average** | **129.7** |

Table S3. Flexural strength and modulus of elasticity of 65% PAM measured and certified by TUV SUD PSB Pte Ltd under ASTM D790-17. Test report number 7191324949-MEC24-AY.

| **Material: 65% PAM** | **Flexural strength (MPa)** | **Modulus of elasticity (GPa)** |
| --- | --- | --- |
| Specimen #1 | 225.9 | 7.7 |
| Specimen #2 | 232.5 | 8.5 |
| Specimen #3 | 218.8 | 8.2 |
| Specimen #4 | 239.9 | 8.2 |
| Specimen #5 | 246.6 | 8.9 |
| **Average** | **232.7** | **8.3** |

**Table S4.** Percentages of water molecules that exist as monomers, dimers, trimers and above at 11% and 20% water content. The percentages of water molecules that exist in non-linear clusters, which is an indication of water-water clustering are also shown in the table. Each number is the average of 5 independently prepared PAM-water systems at the specified water content.

| **Water content (%)** | **Percentage of water molecules in water nanoclusters of various sizes (%)** | | | | | | | |
| --- | --- | --- | --- | --- | --- | --- | --- | --- |
|  | **Monomers** | **Dimers** | **Trimers** | **Tetramers** | **Pentamers** | **Hexamers** | **> Hexamers** | **Non-linear clusters** |
| 11 | 40 | 22 | 11 | 7 | 4 | 3 | 11 | 2 |
| 20 | 26 | 16 | 9 | 4 | 3 | 3 | 28 | 11 |

**Table S5.** The average number of H bonds for water molecules at different water contents. Each number is the average of 5 independently prepared PAM-water systems.

|  | **O_water_-H_PAM_** | **O_PAM_-H_water_** | **N_PAM_-H_water_** | **Total** |
| --- | --- | --- | --- | --- |
| 11%, monomers | 1.96 | 1.75 | 0.23 | 3.94 |
| 11%, all water molecules | 1.55 | 1.44 | 0.13 | 3.11 |
| 20%, all water molecules | 1.16 | 1.0 | 0.07 | 2.23 |

**Table S6.** Averaged diffusion coefficients of all water molecules in the PAM-water networks of 11% and 20% water content at different temperatures. For temperature above 500K, water molecules exhibit normal Brownian diffusion, and for temperatures at 400K and below, water molecules exhibit sub-diffusive behavior.

| **Temperature (K)** | **D (10^-5^ cm^2^/s)** | |
| --- | --- | --- |
|  | **11%** | **20%** |
| 800 | 3.01 | 15.9 |
| 600 | 1.34 | 9.47 |
| 500 | 0.52 | 0.58 |
| 400 | Sub-diffusive regime | |

**Table S7.** Median distances and angles of PAM-water at various water contents. 180° represents an ideal and linear H bond. The average values are obtained from 5 independently prepared systems.

|  | 11 *wt*.% | | | 20 *wt*.% | | |
| --- | --- | --- | --- | --- | --- | --- |
|  | O_PAM_-H_water_ | O_water_-H_PAM_ | N_PAM_-H_water_ | O_PAM_-H_water_ | O_water_-H_PAM_ | N_PAM_-H_water_ |
| Median bond distance (nm) | 0.193 | 0.212 | 0.289 | 0.199 | 0.227 | 0.291 |
| Median bond angle (°) | 155.0 | 108.3 | 102.6 | 152.9 | 105.9 | 100.9 |

**Table S8.** Average counts of PAM–PAM and water–PAM H bonds at varying water contents. Each value represents than from five independently prepared systems.

| Water content (%) | | 0 *wt*.% | 11 *wt*.% | | | 20 *wt*.% |
| --- | --- | --- | --- | --- | --- | --- |
|  |  |  | Undeformed | Stretched (1.5%) | Compressed (75%) |  |
| PAM-PAM H bonds | Number of intrachain | 2767 | 1625 | 1601 | 1561 | 1185 |
|  | Total number (intrachain and interchain) | 5070 | 3455 | 3465 | 3427 | 2734 |
|  | % of intrachain | 55 | 47 | 46 | 46 | 43 |
| PAM-water H bonds | Total number | NA | 3714 | 3682 | 3567 | 5105 |
|  | % of water molecules in interchain water nanoclusters | NA | 95.5 | 94.6 | 96.8 | 96.0 |
| Total number of PAM-PAM and water-PAM | | 5070 | 7211 | 7147 | 6994 | 7839 |
| Density of interchainPAM-PAM and water-PAM H bonds (nm^-3^) | | 5.77 | 13.8 | 13.8 | 13.6 | 11.9 |

References

[1]	J. Brandrup, E. H. Immergut, E. A. Grulke, *Polymer Handbook 4th Edition*, Wiley-Interscience, New York **1999**.

[2]	a) S. Mori, H. G. Barth, in *Size Exclusion Chromatography*, DOI: 10.1007/978-3-662-03910-6_7 (Eds: S. Mori, H. G. Barth), Springer Berlin Heidelberg, Berlin, Heidelberg **1999**, p. 95; b) J. Klein, K.-D. Conrad, *Die Makromolekulare Chemie* **1978**, 179, 1635.

[3]	a) D. Van Der Spoel, E. Lindahl, B. Hess, G. Groenhof, A. E. Mark, H. J. Berendsen, *Journal of computational chemistry* **2005**, 26, 1701; b) S. Pronk, S. Páll, R. Schulz, P. Larsson, P. Bjelkmar, R. Apostolov, M. R. Shirts, J. C. Smith, P. M. Kasson, D. Van Der Spoel, *Bioinformatics* **2013**, 29, 845.

[4]	a) K. Vanommeslaeghe, E. Hatcher, C. Acharya, S. Kundu, S. Zhong, J. Shim, E. Darian, O. Guvench, P. Lopes, I. Vorobyov, *Journal of computational chemistry* **2010**, 31, 671; b) A. D. MacKerell Jr, D. Bashford, M. Bellott, R. L. Dunbrack Jr, J. D. Evanseck, M. J. Field, S. Fischer, J. Gao, H. Guo, S. Ha, *The Journal of Physical Chemistry B* **1998**, 102, 3586.

[5]	M. Huš, T. Urbic, *The Journal of Chemical Physics* **2012**, 136.

[6]	W. Humphrey, A. Dalke, K. Schulten, *Journal of molecular graphics* **1996**, 14, 33.
